# Editorial: Biology and Pathogenesis of *Legionella*

**DOI:** 10.3389/fcimb.2018.00328

**Published:** 2018-09-19

**Authors:** Hayley J. Newton, Elizabeth L. Hartland, Matthias P. Machner

**Affiliations:** ^1^Department of Microbiology and Immunology, University of Melbourne at the Peter Doherty Institute for Infection and Immunity, Melbourne, VIC, Australia; ^2^Centre for Innate Immunity and Infectious Diseases, Hudson Institute of Medical Research, Clayton, VIC, Australia; ^3^Department of Molecular and Translational Science, Monash University, Clayton, VIC, Australia; ^4^Division of Molecular and Cellular Biology, Eunice Kennedy Shriver National Institute of Child Health and Human Development, National Institutes of Health, Bethesda, MD, United States

**Keywords:** *Legionella*, host-pathogen interactions, Dot/Icm effector, intravacuolar pathogen, Legionnaires' disease

*Legionella* species are a large collection of environmental Gram-negative bacteria that have evolved the capacity to replicate to high numbers in a range of eukaryotic cells. This trait enables some *Legionella* to be pathogenic to humans, particularly when the individual is immunocompromised. Since the 1976 outbreak of Legionnaires' disease, and discovery of *Legionella pneumophila* as a human pathogen, this bacterium has been the subject of significant research effort and important scientific discoveries.

Research on *Legionella* has impacted bacteriology, with important studies covering pathogenesis, transcriptional regulation, physiology and metabolism. *Legionella* has also been used as a valuable tool to inform understanding of environmental microbiology, protein biochemistry, innate and adaptive immunology and eukaryotic cell biology. Under the umbrella of this research topic 14 publications have delved into these diverse research areas. These publications highlight the current breadth of *Legionella* research and the future direction of the field of study. Seven original research papers, six reviews and one hypothesis and theory article have contributed to this research topic.

The Dot/Icm type IVB secretion system is essential for virulence of *Legionella*, mediating intracellular survival and establishment of the *Legionella*-containing vacuole (LCV) replicative niche through the collective action of a large cohort of effector proteins. Four original research papers have revealed novel findings regarding effectors of the Dot/Icm system (Allgood et al.; Kubori et al.; Speir et al.; Price et al.).

The Dot/Icm effector SidF has previously been shown to block host cell apoptosis during *L. pneumophila* infection (Banga et al., 2007). Here Speir et al. examined the role of apoptosis during *L. pneumophila* infection and demonstrate a SidF-independent evasion of host cell death. The single-cell live-cell imaging approach developed in this study allows detailed analysis of the dynamics of host viability throughout *L. pneumophila* infection.

Allgood et al. provide further insight into the effector AnkX, previously shown to post-translationally modify the host GTPases Rab1 and Rab35 through the addition of a phosphocholine moiety (Mukherjee et al., 2011). In this study, the functional implications of this enzymatic activity are explored with demonstration that AnkX perturbs endocytic recycling, a Rab35 dependent process. This activity aids *L. pneumophila* infection by inhibiting LCV-lysosome fusion (Allgood et al.). Another effector, RavZ, acts to block host autophagy by irreversibly deconjugating LC3 (Choy et al., 2012). Using a unique *Salmonella-Legionella* co-infection system Kubori et al. demonstrate that RavZ may also target ubiquitin. RavZ interfered with the recruitment of ubiquitin to the *Salmonella*-containing vacuole in a manner that depends on the previously identified catalytic site (Kubori et al.).

RavZ and AnkX are examples of Dot/Icm effectors that act by post-translationally modifying host targets to control their activity. Price et al. explored the opposite phenomenon; host post-translational modification of *Legionella* effectors. Specifically, they investigated factor inhibiting HIF1 (FIH)-mediated asparaginyl hydroxylation, known to impact protein-protein interactions. They demonstrated that the effectors AnkH and AnkB can be hydroxylated by human cells (Price et al.). Components of the modification machinery are recruited to the LCV and removal of FIH leads to increased LCV-lysosome fusion indicating a role for asparaginyl hydroxylation in maintenance of the replicative LCV.

Several original research publications in this research topic explored the importance of specific proteins and processes not linked to the Dot/Icm system (Li and Faucher; Hoppe et al.; Lama et al.). This research represents fundamental knowledge and exciting new targets that could be used to develop novel approaches toward control of urban outbreaks of Legionnaires' disease.

Hoppe et al. demonstrated that PilY1 makes important contribution to *L. pneumophila* virulence. Highly homologous to PilY1 of *Pseudomonas aeruginosa*, the *L. pneumophila* PilY1 is an outer membrane protein contributing to adherence, invasion and replication within different human cells. Given that PilY1 also promotes twitching motility of *L. pneumophila*, future studies may demonstrate that this virulence factor is also crucial for bacterial dissemination during lung infection.

Lama et al. developed a transposon mutagenesis screen to identify *L. pneumophila* mutants attenuated for growth in amoeba. Interestingly, the researchers identified multiple genes required in amoebae but not macrophages and two genes required for *L. pneumophila* replication in both host cells (Lama et al.). The latter two genes, conserved among several human pathogens, both encode components of an ATP binding cassette (ABC) transporter complex of unknown function.

Central to the capacity of *L. pneumophila* to cause disease is its ability to persist within water environments for extended periods of time. Li and Faucher have explored this phenomenon, identifying and characterizing a *L. pneumophila* membrane protein important for survival in water. LasM, *Legionella* aquatic survival membrane protein, has no impact on infectivity of *L. pneumophila* but is required for the culturability of the organism from water (Li and Faucher). Interestingly, homologs of LasM are present in many *Legionella* species and other aquatic bacteria suggesting LasM may represent a common strategy for persistence in aquatic environments.

The *Legionella* life cycle and metabolic adaptation (Oliva et al.), regulation of flagellation (Appelt and Heuner), diversity of protozoan hosts (Boamah et al.), deciphering effector function (Schroeder), manipulation of host ubiquitination (Qui and Luo) and the role of host retrograde trafficking during *Legionella* infection (Bärlocher et al.) are all explored by the collection of reviews presented in this research topic. Together these review articles provide a comprehensive reference that reflects our state of the art understanding of *Legionella*.

Oliva et al. examined the metabolic and morphologic changes that *L. pneumophila* initiates in response to environmental cues. The authors have summarized significant research deciphering how *L. pneumophila* is able to adapt to extracellular and intracellular environments and nutrient availability via the stringent response. This regulatory network allows *L. pneumophila* to transit between a replicative and transmissive form demonstrating that environmental adaptation is an essential trait for virulence (Oliva et al.). Flagellation represents a key morphological trait of the transmissive form of *L. pneumophila*. Appelt and Heuner authored a detailed review of flagellation with particular focus on the regulatory networks that influence this trait. The central importance of motility to *L. pneumophila* virulence is highlighted but also that flagellation is not a universal trait of Legionellaceae, with some pathogenic species remaining non-flagellated (Appelt and Heuner).

Interaction between *Legionella* and their natural protozoan hosts is often overlooked in pathogenesis studies, yet this interaction is central to the evolution and environmental persistence of the species. Boamah et al. present a comprehensive exploration of the natural broad host range of *L. pneumophila* and reflect on the diversity of these interactions which are currently poorly represented in *Legionella* host-pathogen interaction studies.

Strategies toward functional understanding of the extensive cohort of *Legionella* Dot/Icm effectors is reviewed by Schroeder. This is a timely review given that recent comparative genomics studies have revealed the massive number of effectors present within the *Legionella* pangenome (Gomez-Valero et al., 2014; Burstein et al., 2016). Schroeder discusses different approaches, beyond traditional genetics, to uncover effector functions including identification of protein targets and profiling post-translational modifications using newly developed technologies.

Research into the eukaryotic pathways targeted by Dot/Icm effectors has yielded significant insight into novel mechanisms of controlling the eukaryotic cell. Reviews by Qui and Luo and Bärlocher et al. summarize current knowledge of how *Legionella* Dot/Icm effectors modulate the host ubiquitin network and retrograde trafficking respectively. Ubiquitin is intrinsic to many vital eukaryotic cellular processes impacting protein stability, localization and/or interactions. Many *L. pneumophila* effectors are known to manipulate this pathway through both unique actions and functional mimicry of eukaryotic enzymes. The overview of this research area, provided by Qui and Luo, presents a clear demonstration that *L. pneumophila* has the capacity to control all aspects of the host ubiquitin network yet the impact this has on LCV biogenesis remains poorly understood. The review by Bärlocher et al. draws on recently published data to propose the LCV as an acceptor compartment for retrograde transport vesicles. Retrograde trafficking aids in restriction of several intracellular bacterial pathogens and is manipulated by *L. pneumophila* through the functionally undefined effector RidL (Bärlocher et al.).

Finally, a Hypothesis and Theory publication explored the complex ideas behind effector redundancy (Ghosh and O'Connor). Redundancy has been a long-standing hurdle toward revealing the importance of specific effectors during intracellular replication of *L. pneumophila*. This article outlines different types of redundancy that have been uncovered in *Legionella* pathogenesis and the selective pressure that has led to this redundancy.

The scientific snapshot encompassed by this research topic demonstrates that *Legionella* species are mysterious bacteria from which decades of dedicated scientific research has provided significant advances in knowledge. The future application of new technologies and development of new approaches to study *Legionella* will undoubtedly continue to unveil great insights with broad implications.

## Author contributions

All authors listed have made a substantial, direct and intellectual contribution to the work, and approved it for publication.

### Conflict of interest statement

The authors declare that the research was conducted in the absence of any commercial or financial relationships that could be construed as a potential conflict of interest.
